# Preoperative Chronic Pain as a Risk Factor for Early Postoperative Cognitive Dysfunction in Elderly Patients Undergoing Hip Joint Replacement Surgery: A Prospective Observational Cohort Study

**DOI:** 10.3389/fnins.2021.747362

**Published:** 2021-12-17

**Authors:** Xiaorong Huai, Yingfu Jiao, Xiyao Gu, Huichen Zhu, Lingke Chen, Yichen Fan, Weifeng Yu, Diansan Su, Hong Xie

**Affiliations:** ^1^Department of Anesthesiology, The Second Affiliated Hospital of Soochow University, Suzhou, China; ^2^Department of Anesthesiology, Renji Hospital, School of Medicine, Shanghai Jiao Tong University, Shanghai, China

**Keywords:** postoperative cognitive dysfunction, preoperative pain, risk factor, elderly patients, hip joint replacement surgery

## Abstract

**Background:** Although major joint replacement surgery has a high overall success rate, postoperative cognitive dysfunction (POCD) is a common complication after anesthesia and surgery, increasing morbidity and mortality. Identifying POCD risk factors would be helpful to prevent and decrease the occurrence of POCD. We hypothesized that preoperative chronic pain increases the risk of POCD.

**Methods:** A single-center, observational, prospective cohort study was conducted from January 2018 to March 2020. All consecutive elderly patients (>65 years) who underwent elective total hip arthroplasty or hemiarthroplasty with general anesthesia by the same surgeon were enrolled. The patients underwent neuropsychological testing preoperatively and at 7 days and 2 months after surgery. To determine POCD, a nonsurgical control group was recruited from the general community.

**Results:** Of the 141 patients who finished the neuropsychological testing 7 days after surgery, 61 (43.2%) had preoperative chronic pain. Of the 61 patients, 17 (27.9%) developed POCD; of the 79 patients with no chronic pain, 10 (12.7%) had developed POCD by 7 days after surgery. Multivariate logistic regression analysis identified preoperative chronic pain as a risk factor of POCD assessed 7 days after surgery (odds ratio 6.527; *P* = 0.009). There was no significant difference in the POCD incidence 2 months after surgery between patients with and without preoperative chronic pain.

**Conclusion:** Preoperative chronic pain was a risk factor of developing POCD within 7 days after surgery in elderly patients following hip joint replacement surgery.

**Clinical Trial Registration:** [www.ClinicalTrials.gov], identifier [NCT03393676].

## Introduction

Major joint replacement surgery is one of the most common elective procedures and is performed primarily in elderly adults. After major joint replacement surgery, the majority of patients experience substantial relief from both functional disability and pain. Despite the overall success of major joint replacement surgery, patients undergoing this surgical procedure still remain susceptible to cognitive decline, termed POCD, reported rates of which varied between 7% and 75% depending on the definition, assessment tools used, and the population being studied (Deo et al., 2011; Postler et al., 2011). POCD can result in delayed mobilization and discharge from the hospital, worse long-term cognitive dysfunction, and a higher mortality (Rudolph and Marcantonio, 2011).

Several factors have been shown to be risk factors of POCD after hip and knee surgery, including the effects of anesthetics (Duggleby and Lander, 1994; Zywiel et al., 2014), increased age, fewer formal education years or lower reading level, cerebral microemboli caused by fat or marrow entering the blood during surgery (Patel et al., 2010), lower preoperative brain integrity, and lower preoperative executive and memory functions (Monk et al., 2008; Greene et al., 2009; Smith et al., 2009; Price et al., 2014, 2017; Saczynski et al., 2014; Shioiri et al., 2016). However, it is unclear if preoperative pain, which is one of the main reasons of major joint replacement surgery, is also a risk factor of POCD.

The International Association for the Study of Pain defines pain as “an unpleasant sensory and emotional experience associated with actual or potential tissue damage, or described in terms of such damage” (Treede, 2018). The IASP subcommittee on taxonomy defined it in 1986 as “pain without apparent biological value that has persisted beyond the normal tissue healing time usually taken to be 3 months.” Chronic pain was defined as persistent or recurrent pain lasting longer than 3 months (Treede et al., 2015).

It is known that pain directly impairs cognitive function (Price, 2000). Additionally, previous studies have identified the cross-sectional differences in cognition between elderly adults with and without pain (Hart et al., 2000; Moriarty et al., 2011; van der Leeuw et al., 2016). However, no studies have investigated the role of preoperative pain on the occurrence of POCD. In this study, we conducted a single-center, observational, prospective cohort trial in elderly patients who planned to undergo hip joint replacement surgery with general anesthesia to test our hypothesis that preoperative chronic pain is a risk factor of POCD after major joint replacement surgery.

## Materials and Methods

### Study Design

A single-center, prospective, observational cohort study was conducted in Renji Hospital, School of Medicine, Shanghai Jiao Tong University (Shanghai, China), from January 2018 to March 2020. The study was conducted in accordance with the Declaration of Helsinki and was approved by the local ethics committee (RJ198K) (registered at Clinicaltrials.gov: NCT03393676). Written informed consent was obtained from all patients. All investigators were well trained in neuropsychological testing and pain evaluation.

### Patient Selection

All consecutive patients who underwent elective total hip arthroplasty or hemiarthroplasty with general anesthesia performed by a single surgeon were enrolled. The inclusion criteria were as follows: (1) >65 years old, (2) speaks Chinese Mandarin, (3) planned to undergo major low limb surgery such as elective total hip arthroplasty or hemiarthroplasty with general anesthesia, (4) signed the informed consent form, and (5) assessed as ASA classifications I to II.

The exclusion criteria included the following: (1) existing cerebral disease or a history of neurological and psychiatric disease, including psychosis, stroke, epilepsy, and Alzheimer’s disease; (2) existing cognitive impairment as evidenced by the MMSE scores less than 24; (3) severe hearing or visual impairment; (4) unwillingness to comply with the protocol or procedures; (5) inability to communicate in Chinese Mandarin; (6) presence of serious pulmonary, heart, liver, or renal insufficiency; and (7) had undergone anesthesia or surgery within the past 30 days.

To determine POCD and cognitive decline, it is necessary to use a nonsurgical control group (Lewis et al., 2004). The selection criteria of the subject controls were the same, wherein subjects were matched to the elective total hip arthroplasty or hemiarthroplasty replacement surgery sample by age, gender, and education, but they had no chronic pain. The controls were recruited from the general community. The community control group was recruited over the same time frame and underwent neuropsychological testing at the same time intervals, corresponding to assessments in the patients undergoing surgery.

All patients underwent elective total hip arthroplasty or hemiarthroplasty. All clinical care followed routine clinical practice. All surgical plans were decided and performed in a standard manner by the same orthopedic surgeon.

All patients received general anesthesia according to routine clinical practice. Anesthesia was induced using intravenous (IV) midazolam (0.02 mg/kg), propofol (1–2 mg/kg), sufentanil (0.2–0.5 μg/kg), and rocuronium (0.6 mg/kg). Anesthesia was maintained with inhaled sevoflurane (1MAC) and intravenous propofol (2–3 mg/kg/h), remifentanyl (0.2–0.5 μg/kg/min), and rocuronium (10 μg/kg/min). During anesthesia, radial artery was cannulated for intra-arterial pressure monitoring. Electrocardiograph (ECG), PETCO_2_, and end-tidal sevoflurane concentration were monitored during operation. Ephedrine was given if mean arterial blood pressure decreased to less than 75% of baseline. Systolic blood pressure was kept between 80 and 90 mmHg.

All patients received standardized perioperative care, including preoperative and intraoperative care and postoperative pain control. We recorded whether patients used nonsteroidal anti-inflammatory drugs preoperatively or not (Table 1). However, the dose, duration, and frequency of use of nonsteroidal anti-inflammatory drugs were not assessed in our dataset. All patients received nonsteroidal anti-inflammatory drugs 10 min before the end of surgery. Postoperative PCIA was provided by means of a continuous intravenous infusion of sufentanyl (1.5 μg/kg) plus nonsteroidal anti-inflammatory, which was diluted into 100 ml with normal saline. The pump was programmed as a background infusion of 1 ml/h, a PCIA rescue dose of 2 ml, and a lockout period of 15 min. Tramadol was given on request as rescue analgesia postoperatively when patients reported severe pain. This analgesic schedule include opioid medicine that was just maintained for 48 h.

**TABLE 1:** General characteristics of patients.

Characteristic	Total, *n* = 141	Non-chronic pain, *n* = 80 (56.7%)	Chronic pain, *n* = 61 (43.2%)	*P*-value
Age (year)	69.6 (6.2)	69.1 (6.2)	70.1 (6.1)	0.347
Gender (male/female)	60/81	34/46	26/35	1.000
BMI (kg/m^2^)	24.3 (3.7)	23.1 (3.4)	25.9 (3.4)	<0.001
Education Level (year)	9.0 (9.0–12.0)	9.0 (8.0–12.0)	9.0 (6.0–12.0)	0.079
History of hypertension	68 (48.2)	34 (42.5)	34 (55.7)	0.129
History of diabetes	28 (20.6)	16 (20.0)	12 (19.7)	1.000
History of smoking	14 (9.9)	9 (11.2)	5 (8.2)	0.586
ASA grade I/II	57/84	35/45	22/39	0.390
Diagnosis				<0.001
Osteoarthritis	48 (34.0)	2 (2.5)	46 (75.4)	
Femoral neck fracture	84 (59.6)	77 (96.2)	7 (11.5)	
Aseptic necrosis of femoral head	9 (6.4)	1 (1.2)	8 (13.1)	
Surgery type				<0.001
Total hip arthroplasty	86 (61.0)	37 (46.2)	49 (80.3)	
Hemiarthroplasty	55 (49.0)	43 (53.8)	12 (19.7)	
Surgery time (min)	120.0 (90.0–150.0)	111.3 (89.1–150.0)	150.0 (99.6–180.0)	<0.001
Blood loss (ml)	400.0 (200.0–600.0)	400.0 (200.0–600.0)	400.0 (300.0–600.0)	0.312
Blood transfusion (ml)	400.0 (0.0–600.0)	400.0 (0.0–600.0)	400.0 (0.0–700.0)	0.282
Intraoperative hypotension	98 (69.5)	53 (66.3)	45 (73.8)	0.362
Intraoperative ephedrine use	15.2 (13.9)	13.6 (13.3)	17.2 (14.60	0.138
Length of hospital stay (days)	7.0 (7.0–11.0)	7.0 (7.0–9.0)	8.0 (7.0–13.0)	0.014
Chronic pain period (year)	0.0 (0.0–20.0)	0.0 (0.0)	4.3 (5.0)	<0.001
Preoperative nonsteroidal anti-inflammatory drug use	62 (44.0)	32 (40.0)	30 (49.2)	0.307
Postoperative tramadol use	54 (38.3)	29 (36.3)	25 (41.0)	0.603
Postoperative nonsteroidal anti-inflammatory drugs use (7 days)	91 (64.5)	49 (61.3)	42 (68.9)	0.379
Postoperative nonsteroidal anti-inflammatory drugs use (2 months)	19 (14.2)	9 (12.2)	10 (16.7)	0.468
PCIA	107 (75.9)	60 (75.0)	47 (77.0)	0.844
POCD 7 days	27 (19.1)	10 (12.5)	17 (27.9)	0.030
POCD 2 months	10 (7.5)	6 (8.1)	4 (6.7)	1.000

*Values are mean (SD) or median (IQR) or N (%).*

*POCD, postoperative cognitive dysfunction; PCIA, patient controlled intravenous analgesia.*

### Visual Analog Scale

The visual analog scale anchored at 0 and 10 was used to measure pain intensity. Scores of 1–3 were designated as grade 2, scores of 4–7 were designated as grade 3, and scores of 8–10 were designated as grade 4. Patients with a baseline pain of at least 4 on a VAS and lasting for more than 3 months were assigned into the chronic pain group, and the patients without chronic pain were assigned to the non-chronic pain group.

### POCD Identification

We carried out neuropsychological testing, including a battery of six neuropsychological tests at baseline (the day before surgery) and at 7 days and 2 months after surgery. The neuropsychological tests consisted of the MMSE, Visual Reproduction Test, Digit Span Test (forward, backward), Digit Symbol Test, Color Trail Tests (1 and 2), and Stroop Color and Word Test (A, B, and C). All tests were conducted in the same order at each time point. These measures not only are highly sensitive to the types of cognitive impairments but also have no cultural bias.

The MMSE was only used to exclude patients with existing cognitive impairment (MMSE scores < 24) at baseline. The remaining neuropsychological battery of tests addressed three cognitive domains: attentional capacity, executive function, and memory. In the neuropsychological tests including the Visual Reproduction Test, Digit Span Test (forward and backward), Digit Symbol Test, and Stroop Color and Word Test (A, B, and C), the higher scores reflected better performance. The Color Trail Tests (1 and 2) provided estimates of attentional capacity and executive function, where a shorter time reflected better performance.

Since these tests are prone to the test–retest practice effect, an age-matched control group was tested at the same intervals, providing an indication of practice effect with this test battery for the given time intervals between sessions.

Postoperative cognitive dysfunction was defined as a score decrease of 1.96 SDs below baseline in two or more neuropsychological tests or by a decrease of 1.96 SDs in the combined *Z*-score. The *Z*-score was calculated according to the methods of Rasmussen (Lewis et al., 2004). The formula was as follows:


$$\text{Z}=\frac{△X-△{\text{X}}_{\text{control}}}{\mathrm{SD}{\left(△X\right)}_{\text{control}}}$$

△X was obtained from the postoperative score (7 days or 2 months) to the subtracted preoperative score. △X_control_ was calculated from the community controls. SD(△X)_control_ was SD for the changes in test results in the community control group. A combined *Z*-score was calculated as the sum of *Z*-scores divided by the SD for this sum of *Z*-scores in the community control group. Data from the healthy control group were used to gain information on the practice effect and normal distribution in the test results for this age group, and the *Z*-scores were calculated (Rasmussen et al., 2001).

### Statistical Analysis

Continuous data are reported in the tables as mean and SD or as median and interquartile range, and categorical data were reported as frequency and percentage. We examined the demographic and health characteristics per dichotomous pain variable (“chronic pain” vs. “non-chronic pain”). Between-group differences were analyzed by using the analysis of variance or the Mann–Whitney rank sum test for continuous measures and the chi-squared test or Fisher’s exact test for categorical measures. For univariate analysis, we used the Mann–Whitney *U* test for nonnormal distributions or the independent samples *t*-test for normally distributed continuous variables and the chi-square test for categorical variables to compare differences between the group with postoperative POCD and that without postoperative POCD (7 days and 2 months after surgery). All clinically relevant and statistically significant preoperative variables were then entered into a multivariate logistic regression analysis using a forward entry method to identify independent preoperative risk factors for POCD. The Hosmer–Lemeshow goodness of fit test was performed to evaluate model fitting of the logistic multivariable model. Data are presented as the odds ratio and 95% confidence intervals (CIs). Values of *P* < 0.05 were considered to be indicative of statistical significance.

### Sample Size Calculation

Pass (Version 15.0, NCSS, LLC, Kaysville, UT, United States) software was used for the sample size calculation. Logistic regression tests for odds ratios with one binary X procedure were performed. With α = 0.05, a power of 80%, and an odds ratio = 3.0, the POCD incidence of major joint replacement was approximately 20%, and chronic pain prevalence in such patients was at nearly 50% (Wylde et al., 2015; van der Leeuw et al., 2016). We then estimated that a total of 135 patients would be required for the study. To set the attrition rate at 10%, a total of 148 patients were required.

## Results

**FIGURE 1:**
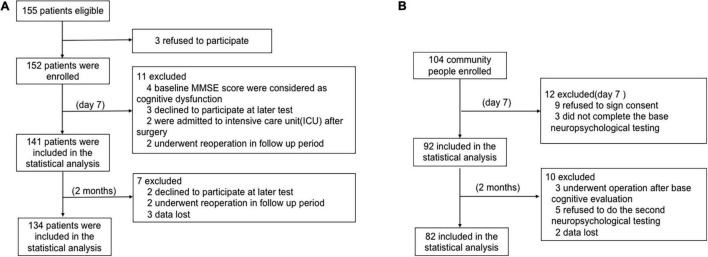
The flow chart of this study. **(A)** Patient flow chart. **(B)** Health community people flow chart.

A total of 155 consecutive patients were enrolled in this study. The trial profile is shown in Figure 1A. Patients were excluded for the following reasons: three refused to sign the informed consent form, four baseline MMSE scores were <24, three declined to participate in a later test, two were admitted to an intensive care unit after surgery, and two underwent reoperation in the follow-up period. The remaining 141 (57.4% female) patients were included in the diagnosis of early POCD at day 7. Additionally, two patients failed to show up for their final evaluation at 2 months, two underwent further surgery prior to follow-up, and three had lost data. Hence, the remaining 134 patients’ cognitive test results were included at 2 months.

Among the 104 age- and education-matched community adults enrolled for the POCD calculations in this study, nine refused to sign the informed consent, three did not complete the baseline neuropsychological testing, three underwent operations after baseline cognitive evaluation, five declined to continue, and two had lost data. Finally, 82 subjects were included in the statistical analysis (Figure 1B).

### Patients’ General Characteristics

Table 1 presents the patients’ general characteristics. At day 7 postoperatively, a total of 27 patients developed cognitive decline, of which 17 had chronic pain preoperatively (27.9%, *P* < 0.05). The BMI of patients with chronic pain was significantly higher than that of those without chronic pain (*P* < 0.05). There was a significant difference in the surgery type, surgery time, and length of hospital stay between patients with chronic pain and patients without chronic pain (*P* < 0.05) (Table 1). No significant difference in PCIA use (77.0 vs. 75.0%) and nonsteroidal anti-inflammatory drugs use before (49.2 vs. 40.0%) or after surgery (7 days and 2 months) between patients with chronic pain and patients without chronic pain was found (Table 1). Table 2 presents the general characteristics of the community controls.

**TABLE 2:** General characteristics of the community people not receiving surgery.

Characteristic	*N* = 82
Age (year)	67.5 (65–82)
Gender (male/female)	29/53
Education level (year)	9.5 (2.7)
History of hypertension	40 (48.8)
History of diabetes	17 (20.7)
History of smoking	6 (7.3)
ASA grade I/II	32/50

*Values are mean (SD) or median (IQR) or N (%).*

### VAS Scores of Patients Before and After Surgery

The VAS scores were significantly higher in patients with chronic pain than in those without chronic pain (*P* < 0.05) before surgery and 7 days after surgery. Patients had similar VAS scores after surgery (3 days and 2 months), regardless of whether they had chronic pain or not (Table 3). There was no significant difference in analgesic use before surgery and after surgery (3 days, 7 days, and 2 months) between patients with chronic pain and those without chronic pain (Table 1). However, VAS score and analgesic use (3 days and 2 months after surgery) of patients with POCD were significantly higher than those of patients without POCD (Tables 4, 5).

**TABLE 3:** VAS scores of the patients.

Time	VAS grade	Non-chronic pain, *n* = 80 (56.7%)	Chronic pain, *n* = 61 (43.2%)	*P*-value
Preoperative	0–2	41 (51.2)	0 (0)	<0.001
	3–4	39 (48.8)	61 (100)	
**Postoperative**				
3 days	0–2	51 (63.7)	35 (57.4)	0.477
	3–4	29 (36.3)	26 (42.6)	
7 days	0–2	79 (98.8)	56 (91.8)	0.044
	3–4	1 (1.3)	5 (8.2)	
2 months	0–2	69 (93.2)	52 (86.7)	0.284
	3–4	5 (6.8)	8 (13.3)	

*Values are N (%). VAS, visual analog scale.*

**TABLE 4:** Logistic analysis for the POCD occurred at 7 days after surgery.

Characteristic	Univariate analysis	Multivariate analysis	
	
	*p*-value	Odds ratio (95% CI)	*P*-value
Preoperative chronic pain	0.030	6.527 (1.583–26.908)	0.009
Postoperative VAS score (3 days)	0.042	1.726 (0.540–5.519)	0.358
Postoperative tramadol use	0.049	1.561 (0.349–6.983)	0.560
Intraoperative hypotension	0.003	6.608 (0.978–44.660)	0.053
Intraoperative ephedrine use	0.001	1.004 (0.961–1.049)	0.850
Pain period	0.136	0.948 (0.832–1.080)	0.424
BMI	0.509	0.862 (0.739–1.006)	0.059
Surgery time	0.977	1.001 (0.993–1.010)	0.749
Diagnosis	0.193	1.311 (0.451–3.813)	0.619
Surgery type	1.000	0.566 (0.188–1.707)	0.312
Length of hospital stay (days)	0.749	1.050 (0.885–1.246)	0.574

*VAS, visual analog scale.*

**TABLE 5:** Logistic analysis for the POCD occurred at 2 months after surgery.

Characteristic	Univariate analysis	Multivariate analysis	
	*p*-value	Odds ratio (95% CI)	*P*-value
Postoperative VAS score (2 months)	0.037	0.514(0.139−1.905)	0.319
Intraoperative hypotension	0.034	0.000	0.998
Intraoperative ephedrine use	0.004	1.047(0.982−1.116)	0.164
Postoperative nonsteroidal anti-inflammatory drug use (2 months)	0.035	29.844(0.813−1095.101)	0.065
Preoperative chronic pain	1.000	1.387(0.152−12.681)	0.772
Pain period	0.567	0.792(0.509−1.232)	0.301
BMI	0.681	1.139(0.929−1.397)	0.211
Surgery time	0.203	0.982(0.962−1.003)	0.099
Diagnosis	0.588	1.126(0.136−9.351)	0.912
Surgery type	1.000	1.870(0.315−11.088)	0.491
Length of hospital stay (days)	0.153	0.992(0.697−1.413)	0.966

*POCD, postoperative cognitive dysfunction; VAS, visual analog scale.*

### Neuropsychological Test Scores of Patients With Chronic Pain and Patients Without Chronic Pain Before and After Surgery

There was significantly (*P* < 0.05) higher scores on the Visual Reproduction Test in patients without chronic pain than in patients with chronic pain before surgery (Table 6). Seven days after surgery, scores on the Visual Reproduction Test and Stroop Color and Word Tests B and C were significantly (*P* < 0.05) higher in patients without chronic pain than in patients with chronic pain (Table 6). Scores on the Stroop Color and Word Test B were significantly (*P* < 0.05) higher in patients without chronic pain than in patients with chronic pain 2 months after surgery (Table 6).

**TABLE 6:** Neuropsychological test scores of patients before and after surgery.

Neuropsychological test scores Mean (SD)	Community people, *n* = 82	Non-chronic pain, *n* = 80 (56.7%)	Chronic pain, *n* = 61 (43.2%)	*P*-value
**Preoperative**				
MMSE	27.6 (1.2)	27.5 (1.6)	27.3 (1.8)	0.541
Digit Span Test	12.4 (2.2)	11.4 (2.2)	11.1 (1.8)	0.339
Digit Symbol Test	32.2 (10.8)	28.9 (11.1)	31.9 (12.7)	0.263
Visual Reproduction Test	8.3 (2.7)	7.8 (2.7)	6.8 (2.9)	**0.045**
Stroop Color and Word Test A	80.7 (15.6)	74.2 (16.9)	70.5 (13.9)	0.169
Stroop Color and Word Test B	64.0 (14.7)	55.4 (14.9)	51.2 (15.2)	0.109
Stroop Color and Word Test C	31.9 (10.0)	28.5 (9.2)	27.3 (8.3)	0.406
Color Trail Tests 1	74.8 (33.9)	81.9 (39.1)	87.6 (31.7)	0.361
Color Trail Tests 2	141.1 (63.1)	148.2 (60.4)	160.9 (49.5)	0.186
**Postoperative (7 days)**				
Digit Span Test	12.8 (2.1)	11.4 (2.2)	10.9 (2.0)	0.119
Digit Symbol Test	36.1 (11.7)	30.4 (12.2)	28.3 (10.3)	0.118
Visual Reproduction Test	8.9 (2.6)	9.0 (2.7)	7.6 (3.0)	0.004
Stroop Color and Word Test A	80.0 (17.0)	71.8 (17.5)	67.8 (14.0)	0.145
Stroop Color and Word Test B	65.3 (14.0)	57.2 (15.2)	52.0 (13.3)	0.034
Stroop Color and Word Test C	34.3 (9.8)	30.3 (8.8)	26.7 (8.5)	0.018
Color Trail Tests 1	74.3 (36.2)	81.0 (42.3)	88.0 (40.2)	0.323
Color Trail Tests 2	128.6 (57.7)	139.6 (61.6)	159.4 (57.4)	0.054
**Postoperative (2 months)**				
Digit Span Test	12.6 (2.1)	11.5 (2.4)	11.4 (2.1)	0.864
Digit Symbol Test	37.4 (12.2)	31.4 (11.8)	28.6 (9.7)	0.177
Visual Reproduction Test	9.2 (2.6)	8.5 (2.9)	7.6 (2.7)	0.089
Stroop Color and Word Test A	72.3 (35.1)	75.7 (16.6)	71.5 (15.1)	0.132
Stroop Color and Word Test B	64.3 (14.2)	58.1 (17.0)	52.7 (13.4)	0.046
Stroop Color and Word Test C	35.2 (10.1)	29.7 (9.9)	28.3 (8.4)	0.371
Color Trail Tests 1	124.8 (62.8)	77.1 (37.7)	83.9 (34.7)	0.288
Color Trail Tests 2	81.8 (16.7)	136.7 (65.4)	154.9 (53.0)	0.085

*Values are mean (SD). MMSE, Mini-Mental State Examination.*

### Patients With Chronic Pain Were More Likely to Develop POCD

**FIGURE 2:**
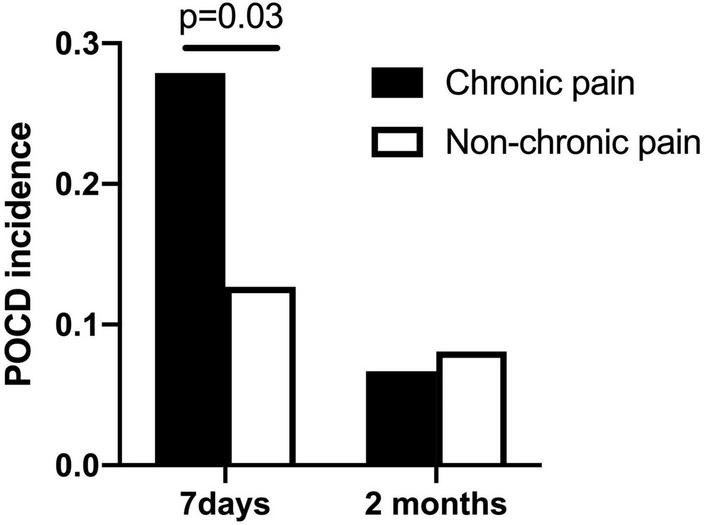
POCD incidence at 7 days and 2 months after surgery (chronic pain patients vs. non-chronic pain patients).

In total, the incidence of early POCD (7 days postoperative) was 27 (19.1%) per 141 patients. The incidence of late POCD (2 months postoperative) was 10 (7.5%) per 134 patients. Among the 141 patients, 61 (43.2%) had chronic pain preoperatively. Of the 61 patients, 17 (27.9%) developed POCD; of the 79 patients without chronic pain, 10 (12.5%) developed POCD at 7 days after surgery (Figure 2).

Among the 134 patients who finished the neuropsychological testing at 2 months after surgery, 60 (44.8%) had chronic pain preoperatively. Of the 60 patients, 4 (6.7%) developed POCD; of the 74 patients without chronic pain, 6 (8.1%) developed POCD at 2 months after surgery.

### Chronic Pain Preoperatively Was a Risk Factor of POCD Within 7 Days After Surgery

In the univariate logistic analysis, we found that preoperative chronic pain (*P* = 0.030), VAS score at 3 days after surgery (*P* = 0.042), postoperative tramadol use (*P* = 0.049), the rate of intraoperative hypotension (*P* = 0.003), and intraoperative ephedrine use (*P* = 0.001) were risk factors of POCD within 7 days after surgery. For multivariable analysis, all the covariates with *P* ≤ 0.10 in the univariate analysis were entered into a backward stepwise logistic regression model for prediction of the primary outcome: incidence of POCD. BMI, diagnosis, surgery type, surgery time, and length of hospital stay were forced into the multivariable model. These variables were selected to account for possible confounding, as there was significant difference in the BMI, diagnosis, surgery type, surgery time, and length of hospital stay between patients with chronic pain and patients without chronic pain (*P* < 0.05) (Table 1). However, in the multivariate logistic analysis, only preoperative chronic pain was a risk factor of POCD within 7 days after surgery (odds ratio 6.527, 95% CI 1.583–26.908, *P* = 0.009; Table 4). There was no difference between the patients who had POCD and those who did not in gender, education, ASA grade, surgery time, blood loss, blood transfusion, length of hospital stay, and VAS scores before or after surgery (day 7).

In the univariate logistic analysis, we found that the VAS score at 2 months after surgery (*P* = 0.037), the rate of intraoperative hypotension (*P* = 0.034), intraoperative ephedrine use (*P* = 0.004), and nonsteroidal anti-inflammatory drug use 2 months postoperation (*p* = 0.035) were risk factors of POCD within 2 months after surgery. In the multivariate logistic analyses, no risk factor for POCD was found within 2 months after surgery (Table 5).

## Discussion

Our study demonstrated that preoperative chronic pain was a potent risk factor (odds ratio 6.527) for POCD within 7 days postoperatively in elderly patients who had undergone major joint replacement surgery but not for POCD at 2 months after surgery. Previous study showed that chronic preoperative pain impaired recovery of attention after surgery in non-elderly patients (Gu et al., 2019). To the best of our knowledge, the present study is the first to identify chronic preoperative pain as a risk factor of POCD in elderly patients.

### Pain of the Patients

The VAS scores indicated that most of the patients without chronic pain had a non-chronic pain lower than grade 3 before surgery while all patients with chronic pain had a grade 3 or 4 preoperative chronic pain. The pain of the patients with chronic pain decreased at 3 days, 7 days, and 2 months after the surgery. As compared with those in patients without chronic pain, the VAS scores were significantly higher in patients with chronic pain at 7 days after surgery (*P* < 0.05) (Table 3).

### Cognition of the Patients

Higher scores on the Visual Reproduction Test in patients without chronic pain than in patients with chronic pain before surgery indicated that patients with chronic pain had poorer abilities in visual memory. However, regarding the corresponding preoperative cognitive abilities, the scores on of Stroop Color and Word Tests B and C (7 days postoperation) were significantly (*P* < 0.05) higher in patients without chronic pain than those with chronic pain, which indicated that patients with chronic pain had poorer of attention and executive function skills, psychomotor processing speed, and concentration. Many studies have demonstrated that chronic pain impaired memory (Hart et al., 2000; Moriarty et al., 2011; Landro et al., 2013; Herbert et al., 2018). Guusje and colleagues found that high-level pain led to cognitive decline and that elderly adults with chronic pain had a higher risk of developing cognitive decline (van der Leeuw et al., 2018). Preoperative chronic pain might affect basic physiological functioning of the brain. Eccleston and colleagues (Baliki et al., 2012) adopted the cognitive–affective theory and proposed that the pain experience demands attention and takes precedence over other attention-demanding cognitive processes. Alternatively, in a demonstration of the competing effects of pain on the brain, it has been reported that the distraction of demanding cognitive tasks led to reduced pain intensity and reduced activation of multiple pain-related brain areas in healthy young and middle-aged adults. Thus, it may be that some elderly persons who have chronic pain are unable to draw their attention away from their pain and thereby have difficulty performing cognitive tasks, while others are able to use distraction to manage their pain (Eccleston and Crombez, 1999; Valet et al., 2004).

### Risk Factors for Postoperative Cognitive Dysfunction

Risk factors for POCD include advanced age, low education level, preexisting cognitive impairment, major surgery, and general anesthesia. Age is the most substantial risk factor. Although there were differences in the BMI, diagnosis, surgery type, surgery time, and length of hospital stay between the patients with chronic pain and patients without chronic pain, these factors showed no difference between the patients with POCD and patients without POCD (7 days and 2 months after surgery). Since there was no difference in the anesthesia treatment, surgery type, surgery time, blood loss, blood transfusion, and PCIA use between the patients with POCD and the patients without POCD (7 days and 2 months after surgery) in our study, it is still unclear whether generalized anesthesia influences the ability of memory and attention in elderly population.

Many factors might contribute to the easier development of POCD in patients with chronic pain. Although there was no difference in major cognitive functioning between patients with and without chronic pain before surgery, cognitive reserve was lower in the patients with chronic pain (Damoiseaux et al., 2008). This finding might be one of the reasons why POCD developed more easily in the patients with chronic pain. However, another reason might be that patients with chronic pain before surgery experience greater intensity of pain. In a meta-analysis of 29,993 patients who had undergone total knee arthroplasty that exclusively examined preoperative risk factors, preoperative pain was most commonly significantly associated with persistent postsurgical pain (Hah et al., 2019). The present study demonstrated that the VAS scores before surgery and 7 days after surgery were significantly higher in the chronic pain group than in the non-chronic pain group. Compared with the patients without early POCD, the patients who developed POCD had significantly higher VAS scores 3 days after surgery and more often requested additional opioids as rescue analgesia postoperatively. Several studies have demonstrated that postoperative pain was associated with the development of POCD (Marino et al., 2009; Chi et al., 2013; Zywiel et al., 2014). Further research is required to understand how preoperative chronic pain affects POCD.

A systematic review of the influence of anesthesia and pain management indicated that general anesthesia may be associated with early POCD, with no effect seen beyond 7 days after joint arthroplasty (Zywiel et al., 2014). And a meta-analysis of 122 studies revealed that peripheral nerve block anesthesia/analgesia use for patients undergoing primary hip and knee arthroplasty (compared with no use) was associated with lower odds ratios for cognitive dysfunction (odds ratio 0.30, 95% CI 0.17–0.53/odds ratio 0.52, 95% CI 0.34–0.80) (Memtsoudis et al., 2021). Multimodal anesthesia protocols have not been definitively demonstrated to reduce the incidence of POCD. Nonopioid postoperative pain management techniques, limiting narcotics to oral formulations and avoiding morphine, have appeared to reduce the risk of POCD (Zywiel et al., 2014).

Chronic pain is very common in patients who undergo major joint surgery. Since patients with chronic pain before surgery are more likely to develop POCD, more attention should be given to this group of patients. Pain management starting in the preoperative period and high-quality anesthesia should be implemented if possible.

This study had some limitations. Individuals with significant cognitive impairment (MMSE < 24) were excluded from our research cohort. Therefore, our results cannot be generalized to elderly persons with moderate to severe cognitive impairment. We did not assess several other factors, such as low postoperative oxygen saturation and inflammatory mediators in blood. Inflammation plays a central role in osteoarthritis pathogenesis. Those factors potentially could have diluted the effect of preoperative chronic pain on the incidence of POCD. In this study, there were more osteoarthritis patients in the chronic pain group than in the non-chronic pain group. Osteoarthritis is a type of autoimmunity disease; thus, it can potentially affect the occurrence of POCD (Wan et al., 2007; Cibelli et al., 2010; Fidalgo et al., 2011; Terrando et al., 2011; Vacas et al., 2013, 2014). In the present study, we did not evaluate the presence of postoperative delirium. Actually, the data analyzed in this study were from the patients who had finished the second neuropsychological test, which indicated that all patients had no delirium at least by day 7 after surgery.

In conclusion, our study revealed that preoperative chronic pain was associated with an increased risk of early POCD in elderly patients after major joint replacement surgery. High-quality perioperative pain management might be helpful to reduce POCD.

## Data Availability Statement

The raw data supporting the conclusions of this article will be made available by the authors, without undue reservation.

## Ethics Statement

The studies involving human participants were reviewed and approved by Renji Ethics Committee. The patients/participants provided their written informed consent to participate in this study. Written informed consent was obtained from the individual(s) for the publication of any potentially identifiable images or data included in this article.

## Author Contributions

WY, DS, and HX conceptualized the study. YJ, WY, and DS designed the study. XH, LC, HZ, and YF conducted the study and collected the data. XH and XG analyzed the data. XH, XG, and DS drafted the manuscript. All authors revised the manuscript, contributed to the article, and approved the submitted version.

## Conflict of Interest

The authors declare that the research was conducted in the absence of any commercial or financial relationships that could be construed as a potential conflict of interest.

## Publisher’s Note

All claims expressed in this article are solely those of the authors and do not necessarily represent those of their affiliated organizations, or those of the publisher, the editors and the reviewers. Any product that may be evaluated in this article, or claim that may be made by its manufacturer, is not guaranteed or endorsed by the publisher.

## Abbreviations

POCD: postoperative cognitive dysfunction
IASP: The International Association for the Study of Pain
ASA: American Society of Anesthesiologists
MMSE: Mini-Mental State Examination
SD: standard deviation
VAS: visual analog scale
PCIA: patient-controlled intravenous analgesia.
